# Supplementary material for: A Food Frequency Questionnaire for Hemodialysis Patients in Bangladesh (BDHD-FFQ): Development and Validation
Source: Nutrients. 2021 Dec 17;13(12):4521. doi: 10.3390/nu13124521 (PMC8707927; doi:10.3390/nu13124521)
Supplement: Supplementary file 1 [file nutrients-13-04521-s001.zip › nutrients-1472644-supplementary.pdf]

Table S1. Food item listing in the final BDHD-FFQ

No.	Food Groupings	Subgroups	Total food items
	Cereals and products	6 subgroups:	17
		Biscuits	
		Cake	
		Bread/ Bun	
		Roti	
		Porota	
		Others	
	Cooked Rice		5
	Meat, Poultry & Products	6 subgroups:	14
		Chicken	
		Beef	
		Mutton	
		Pigeon	
		Egg	
		Internal organs	
	Fish, Shellfish & Products	6 subgroups:	15
		Sea water fish	
		Fresh water fish	
		Small water fish	
		Prawn	
		Dry fish	
		Fish eggs	
	Vegetables	5 subgroups:	7
		Green leafy Vegetable	
		Vegetable	
		Mixed Vegetable	
		Mash	
		Salad	
	Pulses, legumes and their products	2 subgroups:	7
		Lentil	
		Others	
	Milk & Dairy products		10
	Bakery and Sweets		2
	Fruit		13
	Snacks & Finger Foods		16
	Traditional Pitha		3
	[Fast Food Chain		5
	Soup		1
	Noodles and Pasta		1
	Beverages	3 subgroups:	9
		Tea/Coffee	
		Carbonated drinks	
		Other drinks	
	Miscellaneous		7
		**Total food items**	**132**

Table S2. Snapshot of the BDHD-FFQ Form

সাব্জেক্ট কোড (Subject Code):

ক্রমিক নাম্বার [No.]	খাদ্য তালিকা [Food]	খেয়েছেন (হ্যা/না) [Taken (yes/no)]	প্রস্তুত প্রণালী [Preparation method]	ফ্রিকুয়েন্সি [Frequency]				পরিমাপ [Household measurements]	পরিমাণ [Portion taken]	অতিরিক্ত ঝোল (১ টেবিল চামচ) [Extra gravy (1 tbsp)]	নোট [Notes]
				প্রতি দিন [per day]	প্রতি সপ্তাহে [per week]	প্রতি মাসে [per month]	খুব কম [rarely]				
**সিরিয়াল এবং সিরিয়াল জাতীয় খাদ্য [Cereals and products]**											
**A**	**বিস্কুট [Biscuits]**										
1	নোনতা বিস্কুট (যেমন: টিপ, বেকারী) [Salty (Eg: Tip/Bakery)]							১ পিস[1 piece]			
2	মিষ্টি বিস্কুট (যেমন: এনার্জী প্লাস, বেকারী) [Sweet biscuit (Eg: Energy Plus/ Bakery)]							১ পিস [1 piece]			
3	টোস্ট বিস্কুট [Toast Biscuits]							১ পিস [1 piece]			
**B**	**কেক্ [Cake]**										
4	কেক্ [Cake]							১ পিস [1 piece]			
5	ড্রাই কেক্ [Dry cake]							১ পিস [1 piece]			
**C**	**পাউরুটি/ বন [Bread/ Bun]**										
6	পাউরুটি [Bread]							১ পিস [1 piece]			
7	বন [Bun]							১ পিস [1 piece]			
**D**	**রুটি [Roti]**										
8	নানরুটি/ তান্দুরি রুটি [Nan/ Tandoori Roti]							১ পিস [1 piece]			
9	আটার রুটি/ চালের রুটি [Chapati/ Rice flour Roti]							১ পিস [1 piece]			

**E**	**পরটা [**Porota]										
10	পরটা **[**Porota]							১ পিস [1 piece]			
11	লুচি **[**Luchi]							১ পিস [1 piece]			
12	বাখরখানি **[**Bakarkhani]							১ পিস [1 piece]			
**F**	**অন্যান্য [**Others]										
13	নিমকি [Nimki]							১ পিস [1 piece]			
14	মুড়ি / চিড়া ভাজা<br>[Puffed Rice/ Rice flaked fry]							ছোট ১ বাটি<br>[1 s.bowl]			
15	চানাচুর<br>[Chanachur]							ছোট ১ বাটি<br>[1 s.bowl]			
16	চানাচুর মুড়ি মিক্স<br>[Chanachur with Puffed rice Mixed]							ছোট ১ বাটি<br>[1 s.bowl]			
17	চিড়া ভেজা সাথে কলা, দুধ, চিনি<br>[Rice flaked with banana, milk, sugar]							ছোট ১ বাটি<br>[1 s.bowl]			
**ভাত [**Cooked Rice]											
18	সাদা ভাত [Cooked rice]							মিডিয়াম ১ বাটি<br>[1 m.bowl]			
19	পোলাও/ফ্রাইড রাইস<br>[Polao/Fried Rice]							মিডিয়াম ১ বাটি<br>[1 m.bowl]			
20	খিচুড়ি [Hotchpotch]							মিডিয়াম ১ বাটি<br>[1 m.bowl]			

21	বিরিয়ানী [Biryani]							মিডিয়াম ১ বাটি [1 m.bowl]			
22	দুধভাত সাথে কলা বা আম, চিনি [Cooked Rice with Cow's Milk, banana/Mango, sugar]							মিডিয়াম ১ বাটি [1 m.bowl]			

সাব্জেক্ট কোড (Subject Code):

ক্রমিক নাম্বার [No.]	খাদ্য তালিকা [Food]	খেয়েছেন (হ্যা/না) [Taken (yes/no)]	প্রস্তুত প্রণালী [Preparation method]	ফ্রিকুয়েন্সি [Frequency]				পরিমাপ [Household measurements]	পরিমাণ [Portion taken]	অতিরিক্ত ঝোল (১ টেবিল চামচ) [Extra gravy (1 tbsp)]	নোট [Notes]
				প্রতি দিন [per day]	প্রতি সপ্তাহে [per week]	প্রতি মাসে [per month]	খুব কম [rarely]				
**মাংস ও মাংস জাতীয় খাবার** [Meat, Poultry & Products]											
23	মুরগীর মাংস [Chicken]		ফ্রাই/ঝাল ফ্রাই/গ্রিল/রোস্ট [Fried/Jhal Fried/grilled/roast]					১ ম্যাচবক্স [1 matchbox]			
24			কারি/ভুনা [Curry/gravy]					১ ম্যাচবক্স [1 matchbox]			
25			মাংস সাথে সবজী (আলু, পেঁপে, কঁদু) তরকারি [Vegetable (Potato, papaya, Teasle gourd) curry]					ছোট ১ বাটি [1 s.bowl]			
26	গরুর মাংস		কাবাব/হাঁড়ি কাবাব [Kabab/Handi kabab]					১ পিস [1 piece]			

27	[Beef]		কারি/ভুনা [Curry/gravy]					১ ম্যাচবক্স [1 matchbox]			
28			মাংস সাথে সবজী (আলু, পেঁপে, কঁদু) তরকারি [Vegetable (Potato, Papaya, Lau) curry]					ছোট ১ বাটি [1 s.bowl]			
			নেহেরী/নলা [Beef bone (Noli)]					১ ম্যাচবক্স [1 matchbox]			
29	ছাগল মাংস [Mutton]		কারি/ভুনা [Curry/gravy]					১ ম্যাচবক্স [1 matchbox]			
30	কবুতর মাংস [Pigeon]		কারি/ভুনা [[Curry/gravy]]					১ ম্যাচবক্স [1 matchbox]			
31			কবুতর সাথে সবজী (আলু, পেঁপে, কঁদু) তরকারি [Vegetable (Potato, Papaya, Lau) curry]					ছোট ১ বাটি [1 s.bowl]			
32	ডিম [Egg]		পোঁচ/অমলেট/সিদ্ধ/ভুনা [Poach/omlet/boiled/ gravy]					১ পিস [1 piece]			
33			ডিম সাথে সবজী তরকারি [Boil with Vegetable Curry]					ছোট ১ বাটি [1 s.bowl]			
34			ডিমের সাদা অংশ (কুসুম ছাড়া) [Egg white boiled]					১ পিস [1 piece]			
35	ইন্টারনাল অরগানস গরু/মুরগী [Internal Organs Beef/chicken]		কারি/ভুনা [Curry/gravy]					১ ম্যাচবক্স [1 matchbox]			
**মাছ ও সামুদ্রিক মাছ জাতীয় খাবার** [Fish, Shellfish & Products]											
36	সামুদ্রিক মাছ [Sea fish]		ভুনা/ভাজি [Gravy/Fried]					১ ম্যাচবক্স [1 matchbox]			

37	(রুপচাদা/ ফলিমাছ/ ইলিশ/ লইট্টা/ বাটা/ তাপসী/পোহা/ মেনী/টুনা) [Rupchanda/ Foli/ Hilsha/ Loitta/ Bata/Toposi/ poa/ Meni/ tuna]		মাছ সাথে সবজী তরকারি [Fish with Vegetable curry]					ছোট ১ বাটি [1 s.bowl]			
38	নদী/পুকুরের বড় মাছ [Fresh water Fish]		ভুনা/ভাজি [Gravy/Fried]					১ ম্যাচবক্স [1 matchbox]			
39	(বোয়াল/ পাঙ্গাস/ কাতাল/ রুই/ কার্প/ তেলাপিয়া/ স্বরপুটি) [Boal/Pangas/Katla/Ruhi/ Carp/Telapia/Shor puti/Bele]		মাছ সাথে সবজী তরকারি [Fish with Vegetable curry]					ছোট ১ বাটি [1 s.bowl]			
40	নদী/পুকুরের মাঝারি মাছ [Fresh water Fish]		ভুনা/ভাজি [Gravy/Fried]					১ ম্যাচবক্স [1 matchbox]			
41	(কেই/ টাকি/ মাগুর/ শোল/ শিং/ টেংরা/ আইর) [Puti/Mola/Kachki/ Chapila/Kajuli/ Batashi]		মাছ সাথে সবজী তরকারি [Fish with Vegetable curry]					ছোট ১ বাটি [1 s.bowl]			

Table S3. Patient Characteristics in Phase I

Characteristics (n = 87)		n (%)	Mean ± SD
Gender	Male	39 (44.8)	
	Female	48 (55.2)	
Age (years)			50.8±13.1
Body Mass Index (kg/m²)			24.7± 5.7
Dialysis frequency (weekly)	3 times per Week	40 (46.0)	
	2 times per Week	47 (54.0)	
Duration of dialysis (hours)			3.8±0.3
Dialysis vintage (month)			35.7±25.6
Post-dialysis weight (kg)			61.4±13.5
Height (cm)			157.9±9.99

Note: Data are presented as mean ± SD unless stated as percentage (%).

Table S4. Item-Content Validity Index (I-CVI) of the specialists (n=12)

	SPE 1	SPE 2	SPE 3	SPE 4	SPE 5	SPE 6	SPE 7	SPE 8	SPE 9	SPE 10	SPE 11	SPE 12
**FG 1**	4.2	5	4.4	4.4	4.6	4.8	4.2	4.6	4	4.6	4.4	4.4
**FG 2**	4.6	5	4.4	4	5	4.4	4.2	4.6	3.8	4.2	4.6	4.4
**FG 3**	3.8	4.8	4.2	4	4.8	4.8	4.2	4.8	3.8	4.2	4.4	4.2
**FG 4**	4	4.8	4.4	4.6	4.8	4.4	4	4.6	3.8	4.2	4.6	3.8
**FG 5**	4	5	4.6	4.4	4.8	4.8	4	4.6	3.8	4.6	4.4	4.2
**FG 6**	4	4.6	4	4.6	5	5	3.8	4.4	3.8	4.4	4.6	4.2
**FG 7**	4.6	5	5	4.4	5	4.8	4	4.4	3.8	4.8	4.6	3.8
**FG 8**	4	5	4.6	4	5	4.8	4.2	4	3.8	4.2	4.8	4
**FG 9**	4.2	5	4.4	4.4	5	4.6	4	4.4	4	4.2	4.4	4
**FG 10**	4	5	4.8	4	5	4.4	4	4.2	3.8	4.2	4.6	4.2
**FG 11**	4.2	4.6	4.6	4	4.8	4.2	4.2	4.2	3.8	4.2	4.8	4
**FG 12**	4	5	4.8	4.6	5	4.4	4	4	3.8	4.2	4.4	4.6
**FG 13**	3.6	5	4.6	4	5	4.4	4.2	4.2	3.8	4.2	4.8	4
**FG 14**	4	5	4.4	4	5	4.6	4.2	4	3.8	4.2	4.6	4
**FG 15**	4	5	4	4.8	5	4.6	4.2	4.8	3.8	4.8	4.2	4.4
**FG 16**	3.8	5	4.4	4	5	5	4.2	4.2	4	4.2	4.4	4.2
**I-CVI**	0.81	1	1	1	1	1	0.93	1	0.19	1	1	0.87
**S-CVI/Ave Score 0.9**												

Abbreviations: SPE: Specialist; FG: Food Group. S-CVI/Ave: Scale-Content Validity Index based on the average method

Table S5. Summary of comments from the specialists and amateurs

No.	Commenter	Open comments
1	Nutritionist 1	Before collecting data some measurements tools can be demonstrated so that, patients can recall the approximate exact portion of food eaten.
2	Nutritionist 2	Portion size can be written in gram/milliliter as well to understand one small bowl means what etc.
3	Nephrologist 1	Details and looks good.
4	Nephrologist 2	The layout is convenient, and the food items are relevant to local foods.
5	Patient 1	The questionnaire is clear and easy to understand, but it can consider increasing the font size as the dialysis elderly patients with compromised vision could face difficulty reading this FFQ
6	Patient 2	Typically consumed food items are available in BDHD-FFQ, that aid in recalling food intake.

Table S6. Identification of mis-reporters according to EI: BMR Category

EI:BMR Category	3DDR (n=100)	BDHD-FFQ (n=100)
Under-reporter	3 (3%)	--
Acceptable-reporter	97 (97%)	100 (100%)
Over-reporter	--	--

Table S7. Statistical test outcomes and interpretations for nutrient intake.

**Nutrients (Unit)**	**Correlation [a]**		**Paired t-test**	**Percent difference**	**Cross-classification [Opposite tertile (%)]**	**Weighted Kappa**	**Bland Altman-% in Level of Agreement**
	**Gross**	**Energy adjusted**					
Facet of validity	Strength & direction		Agreement	Agreement (size & direction of error)	Agreement (including chance)	Agreement (excluding chance)	Limit of Agreement
Level of validation	Individual		Group	Group	Individual	Individual	Group
Energy (Kcal)	0.67	-	<0.01	18.4	2.06	0.43	96.9%
Protein (g)	0.48	0.26	<0.01	18.3	7.22	0.36	91.8%
Carbohydrate (g)	0.58	0.38	<0.01	17.8	4.12	0.30	96.9%
Fat (g)	0.48	0.25	<0.01	20.0	4.12	0.32	95.8%
Sodium (mg)	0.38	0.34	<0.01	21.2	5.15	0.24	95.8%
Calcium (mg)	0.41	0.28	<0.01	11.0	5.15	0.21	93.8%
Iron (mg)	0.31	0.23	<0.01	33.9	5.15	0.12	95.8%
Potassium (mg)	0.40	0.49	<0.01	18.8	7.22	0.23	96.9%
Phosphate (mg)	0.44	0.53	<0.01	24.6	3.09	0.30	94.8%

[a] Intraclass correlation for gross nutrients; Green color represents "Good", Yellow denotes "acceptable", and Red represents "poor" outcome.

Interpretation criteria for statistical tests (Lombard et al, 2015)

Paired t test: Good: p > 0.05; Poor: ≤ 0.05

Percentage difference: Good: 0.0 – 10.9%; Acceptable: 11.0 – 20.0%; Poor: > 20.0%

Correlations coefficient: Good: ≥ 0.50; Acceptable: 0.20 – 0.49; Poor < 0.20

Cross-classification (% in opposite tertile): Good: ≤ 10%, Poor: > 10%

Weighted Kappa statistics: Good: ≥ 0.61; Acceptable: 0.20 – 0.59; Poor: < 0.20

Bland-Altman - % in LOA: Expected that 95% differences are within the 95% limit of agreement. (Myles et al., 2007)
